# Critical Current Density and Vortex Dynamics in Pristine and Irradiated KCa_2_Fe_4_As_4_F_2_

**DOI:** 10.3390/ma14185283

**Published:** 2021-09-14

**Authors:** Sunseng Pyon, Soichi Taya, Yuto Kobayashi, Ayumu Takahashi, Wenjie Li, Toshihiro Taen, Teng Wang, Gang Mu, Hisashi Kitamura, Ataru Ichinose, Tsuyoshi Tamegai

**Affiliations:** 1 Department of Applied Physics, The University of Tokyo, 7-3-1 Hongo, Bunkyo-ku, Tokyo 113-8656, Japan; taya@issp.u-tokyo.ac.jp (S.T.); momiji3.momo3.keyaki3@gmail.com (Y.K.); naotoshikazuto@gmail.com (A.T.); liwenjie@g.ecc.u-tokyo.ac.jp (W.L.); tamegai@ap.t.u-tokyo.ac.jp (T.T.); 2 Institute for Solid State Physics, The University of Tokyo, Kashiwa 277-8581, Japan; taen@issp.u-tokyo.ac.jp; 3 State Key Laboratory of Functional Materials for Informatics, Shanghai Institute of Microsystem and Information Technology, Chinese Academy of Sciences, Shanghai 200050, China; wangteng@mail.sim.ac.cn; 4 National Institute of Radiological Sciences, National Institutes for Quantum and Radiological Science and Technology, Anagawa, Chiba-shi 263-8555, Japan; mugang@mail.sim.ac.cn; 5 Central Research Institute of Electric Power Industry, Grid Innovation Research Laboratory, 2-6-1, Nagasaka, Yokosuka-shi 240-0196, Japan; kitamura.hisashi@qst.go.jp (H.K.); ai@criepi.denken.or.jp (A.I.)

**Keywords:** iron-based superconductors (IBS), vortex dynamics, ion irradiation, KCa_2_Fe_4_As_4_F_2_

## Abstract

We report the critical current density (*J*_c_) and vortex pinning properties in single crystals of a novel iron-based superconductor (IBS) KCa_2_Fe_4_As_4_F_2_ with large *J*_c_ in the pristine state, before and after introduction of artificial defects by swift-particle irradiation. The effects of 2.6 GeV U and 3 MeV proton irradiations in KCa_2_Fe_4_As_4_F_2_ single crystals on transition temperature *T*_c_ and *J*_c_, including its dose dependence, are systematically studied. *J*_c_~8 MA/cm^2^ under a self-field at 2 K in the pristine crystal is strongly enhanced up to 19.4 and 17.5 MA/cm^2^ by irradiation of 2.6 GeV U-ions and 3 MeV protons, respectively. Suppression of *T*_c_ and dose dependence of *J*_c_ in KCa_2_Fe_4_As_4_F_2_ is different from that in a representative IBS of (Ba,K)Fe_2_As_2_, which can be explained by considering the presence of embedded defects in pristine KCa_2_Fe_4_As_4_F_2_. The vortex dynamics in the pristine and proton irradiated KCa_2_Fe_4_As_4_F_2_ single crystals are also investigated from the analyses of the field dependence of *J*_c_ and the normalized magnetic relaxation rate. In addition to the contribution of embedded defects, weak collective pinning is considered for comprehensive analyses. Vortex dynamics in KCa_2_Fe_4_As_4_F_2_ is similar to those in (Ba,K)Fe_2_As_2_ to some extent, and different from that in anisotropic Li_0.8_Fe_0.2_OHFeSe. Large anisotropy, due to the presence of insulating blocking layers in KCa_2_Fe_4_As_4_F_2_, which leads to much lower irreversibility field (*H*_irr_) compared with 122-type IBSs, strongly affect the vortex dynamics.

## 1. Introduction

Iron-based superconductors (IBSs) are attracting a great deal of attention due to their notable features, such as their relatively high transition temperature (*T*_c_), high upper critical field (*H*_c2_), and large critical current density (*J*_c_) [1]. In typical IBSs, such as 122-type compound (Ba,K)Fe_2_As_2_ [2], electronic states are weakly anisotropic, although their crystal structures are characterized by the presence of two-dimensional Fe*Pn* (*Pn*: As, P) or Fe*Ch* (*Ch*: S, Se, Te) layers. This is in contrast to quasi two-dimensional electronic states in cuprate superconductors with two-dimensional CuO_2_ layers [3,4]. Recently, however, it was suggested that quasi two-dimensional electronic states emerge in novel IBSs, such as 12442-type compounds [5,6,7,8,9] or some IBSs consisting of FeSe layers sandwiched by thick insulating layers [10,11]. The 12442-type IBSs have double Fe_2_As_2_ conducting layers between two neighboring Ca_2_F_2_ insulating layers [5]. Due to the presence of insulating layers, anisotropic electronic states are realized as revealed by the highly anisotropic *H*_c2_ [12,13] and torque analyses [14]. KCa_2_Fe_4_As_4_F_2_ is the first and a well-studied 12442-type IBS [8] and its anisotropic physical properties have been demonstrated. In KCa_2_Fe_4_As_4_F_2_, quasi two-dimensional electronic behavior, which is similar to that of cuprate superconductors, has been revealed by neutron spin resonance [15]. The quasi two-dimensional electronic state was also suggested from the large anisotropy of electrical resistivity, with *ρ_c_*/*ρ_ab_* > 100, and semiconductor-like *ρ_c_* [16]. Furthermore, a large anisotropy parameter, *γ* (= *H*_c2_*^ab^*/*H*_c2_*^c^*) ~8, was also evaluated near *T*_c_ [12,16]. Notable features of this highly anisotropic KCa_2_Fe_4_As_4_F_2_ are moderate *T*_c_ of ~34 K, and larger *J*_c_, and *H*_c2_ compared with 122-type IBSs. Large *H*_c2_//*ab* above 700 kOe at low temperatures has been evaluated [17], although the irreversibility field (*H*_irr_), which separate zero and finite *J*_c_ regions, is relatively low [12,16]. The in-plane *J*_c_ evaluated from the measurement of irreversible magnetization at 2 K under the self-field is 8.2 MA/cm^2^, which is significantly larger compared with that of 122-type IBSs [16]. 

One of the most intriguing topics in KCa_2_Fe_4_As_4_F_2_ is how the significantly high *J*_c_ is enhanced by adding artificial pinning centers. It is well known that *J*_c_ in superconductors, such as cuprates and IBSs, can be enhanced by introducing defects using swift particle irradiations, and significant effects on physical properties, such as remarkable enhancements of *J*_c_ by irradiations of heavy ions and protons have been demonstrated [16,18,19,20,21,22,23,24,25,26,27,28,29,30]. For KCa_2_Fe_4_As_4_F_2_, enhancement of *J*_c_ up to 19 MA/cm^2^ at 2 K under self-field is also demonstrated by introducing columnar defects in terms of heavy-ion irradiation, although the irradiation dose is limited [16]. It is also well known that introducing point defects by proton irradiation is effective at enhancing *J*_c_ in cuprate superconductors [31,32] and IBSs [24,27]. A systematic study of the effect of columnar and point defects on *J*_c_ in KCa_2_Fe_4_As_4_F_2_ is demanded. 

Furthermore, investigation of vortex pinning properties in KCa_2_Fe_4_As_4_F_2_ is also required to understand its *J*_c_ behavior. It is needless to say that vortex dynamics is one of the central issues related, not only to basic solid-state physics, but also to applications. Vortex dynamics in cuprates have been extensively studied in past decades [33,34]. When the *c*-axis coherence length, *ξ_c_*, is larger than the layer distance, *d*, such as in YBa_2_Cu_3_O_7_, the magnetic field generates well-connected quantized vortices via interlayer coupling, which creep collectively when the thermal energy is stronger than the pinning energy [33,34]. On the other hand, when the interlayer coupling is very weak, with *ξ_c_* < *d*, such as in Bi_2_Sr_2_CuCa_2_O_8 + *y*_, quantized vortices can be considered as stacks of pancake vortices, which can creep individually in each layer [33,34]. These results suggest that anisotropic crystal structure and electronic structure strongly affect the behavior of vortices. Vortex dynamics in IBSs has also been studied by comparing them with those in cuprate superconductors. For analyses of vortex dynamics, magnetic relaxation in clean single crystals, where a critical state is realized in the whole sample, has been utilized since transport measurements are challenging due to the extremely large *J*_c_. Up to now, 122-type compounds, such as (Ba,K)Fe_2_As_2_ or Ba(Fe,Co)_2_As_2_, and 11-type compounds, such as Fe(Te,Se) and FeSe, have mainly been studied [24,27,35,36]. In these weakly anisotropic materials with an anisotropy parameter 2–3, vortex pinning properties have been interpreted in either weak-collective pinning or strong pinning scenarios [1,2,24,27,35]. On the other hand, in one of IBSs, Li_0.8_Fe_0.2_OHFeSe, with a highly anisotropic crystal structure, breakdown of conventional creep theory has been pointed out [37]. To understand general characteristics of vortex pinning in anisotropic high temperature superconductors, including cuprates and IBSs, further investigation of vortex dynamics in anisotropic materials, such as KCa_2_Fe_4_As_4_F_2_, is demanded. 

In this study, we investigated the effect of columnar defects and point defects created by irradiation of U ions and protons in KCa_2_Fe_4_As_4_F_2_ single crystals. Changes in *T*_c_ and *J*_c_ were systematically studied as a function of the irradiation dose. We also investigated the vortex pinning properties and the vortex dynamics in KCa_2_Fe_4_As_4_F_2_ before and after the introduction of point defects. A part of the data of KCa_2_Fe_4_As_4_F_2_ were compared to those of (Ba,K)Fe_2_As_2_, which belong to 122-type IBSs and is a representative IBS, and those of Li_0.8_Fe_0.2_OHFeSe, which has anisotropic crystal structure similar to KCa_2_Fe_4_As_4_F_2_.

## 2. Experimental Methods

Single crystals of KCa_2_Fe_4_As_4_F_2_ were grown using the self-flux method with KAs as the flux. Details of sample growth and the basic physical properties are reported in Reference [12]. Ba_0.6_K_0.4_Fe_2_As_2_ single crystals, which were used as reference materials to be compared with KCa_2_Fe_4_As_4_F_2_, were also synthesized using the FeAs self-flux method [27,38]. The 2.6 GeV U ions were irradiated parallel to the *c*-axis at room temperature at RIKEN Ring Cyclotron in RI Beam Factory, operated by RIKEN Nishina Center and CNS, the University of Tokyo. The irradiation dose was evaluated using the dose-equivalent magnetic field called the “matching field”, at which all defects are occupied by single vortices, *B*_Φ_ = *n*Φ_0_. Here, *n* is the areal density of CDs and Φ_0_ is a flux quantum. The 3 MeV protons were irradiated parallel to the *c*-axis at room temperature at the National Institute of Radiological Sciences Heavy Ion Medical Accelerator in Chiba, Japan (NIRS-HIMAC). Cross-sectional observations of the single crystals were performed with a scanning transmission electron microscope (STEM) (JEOL, JEM-2100F, Akishima, Tokyo, Japan). The spatial resolution of the JEOL JEM-2100F microscope was 0.2 nm; however, we set it as 0.5 nm to add contrast to the STEM image. The specimens for STEM were prepared by digging and milling using a focused-ion beam (FIB), which is called the microsampling technique. Final milling using FIB was conducted at an acceleration voltage of 30 kV and with a very weak ion current of ~10 pA without tilting the specimen. Magnetization was measured in a commercial superconducting quantum interference device magnetometer (MPMS-XL5, Quantum Design, San Diego, CA, United States) with an applied magnetic field parallel to the *c*-axis. In this system, temperature accuracy was ±1%, and magnetic field resolutions were 0.1 G up to 5 kOe and 1 G up to 50 kOe.

## 3. Experimental Results and Discussions

In this section, first, the effects of 2.6 GeV U and 3 MeV proton irradiations on *T*_c_ and *J*_c_ estimated from magnetization measurements in KCa_2_Fe_4_As_4_F_2_ are discussed. STEM images of both the pristine and 2.6-GeV U-irradiated KCa_2_Fe_4_As_4_F_2_ are also presented. Second, the vortex dynamics of the pristine and 3 MeV proton irradiated crystals are discussed based on magnetization measurements and analyses of magnetic relaxation.

### 3.1. Effects of 2.6 GeV U and 3 MeV Proton Irradiations on T_c_ and J_c_ in KCa_2_Fe_4_As_4_F_2_

**Figure 1:**
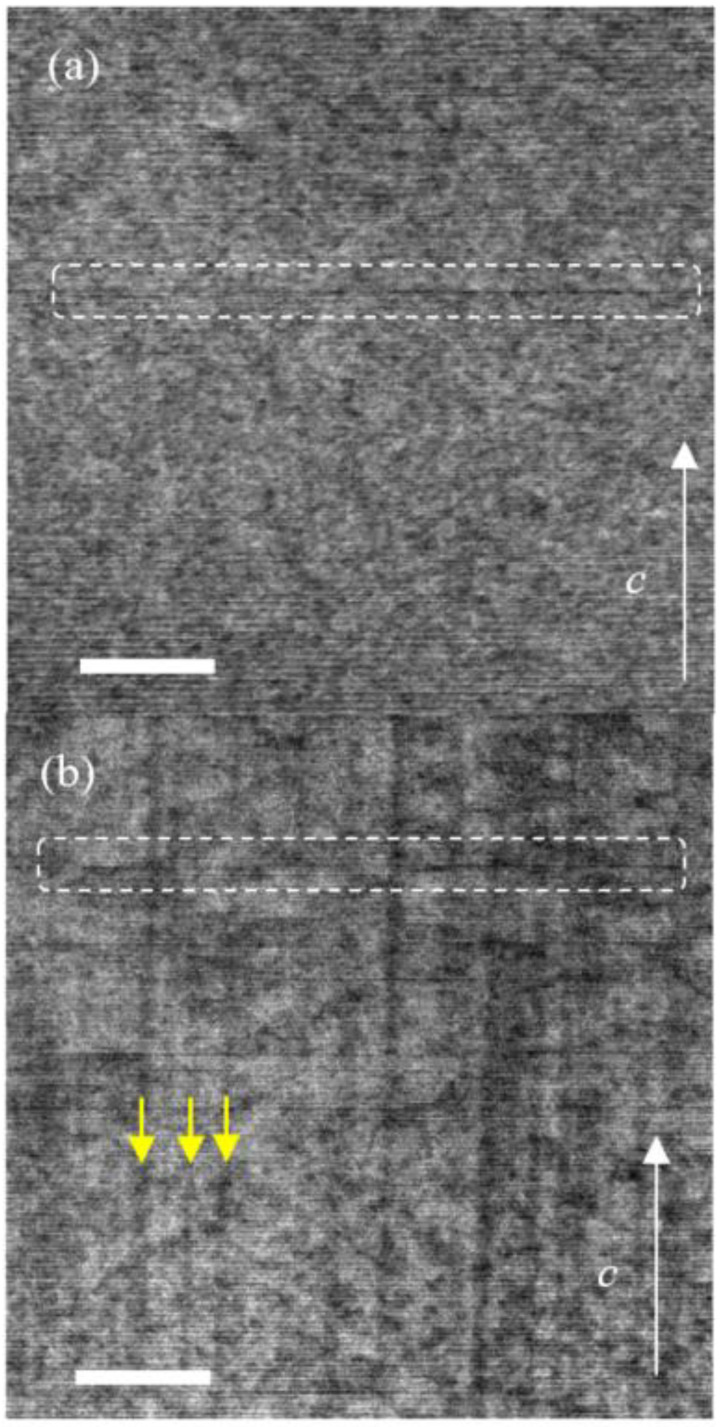
STEM images of (**a**) the pristine and (**b**) 2.6-GeV U-irradiated KCa_2_Fe_4_As_4_F_2_ for an electron beam injected along the *a* axis. Scale bars in (**a**,**b**) correspond to 50 nm. Broken squares in (**a**,**b**) emphasize the location of horizontal black lines in STEM images, which we interpreted to be thin planar defects. Yellow arrows in (**b**) show examples of columnar defects generated by 2.6-GeV U-irradiation.

First, STEM analyses of the pristine and 2.6-GeV U ion-irradiated KCa_2_Fe_4_As_4_F_2_ crystals with an electron beam injected along *a*-axis were performed. As shown in the STEM images in Figure 1a,b, some black lines parallel to the *ab*-plane were observed in both pristine and U-irradiated KCa_2_Fe_4_As_4_F_2_. Although the existence of these sparsely distributed defects cannot explain the significantly larger *J*_c_ in pristine KCa_2_Fe_4_As_4_F_2_, they suggest the presence of atomic scale defects or chemical inhomogeneities, which may have contributed as pinning centers. In the 2.6-GeV U-irradiated KCa_2_Fe_4_As_4_F_2_, clear columnar defects with a diameter ~5 nm along the *c*-axis were observed, which were similar to the case of Ba(Fe,Co)_2_As_2_ and (Ba,K)Fe_2_As_2_ [20,23,39]. 

**Figure 2:**
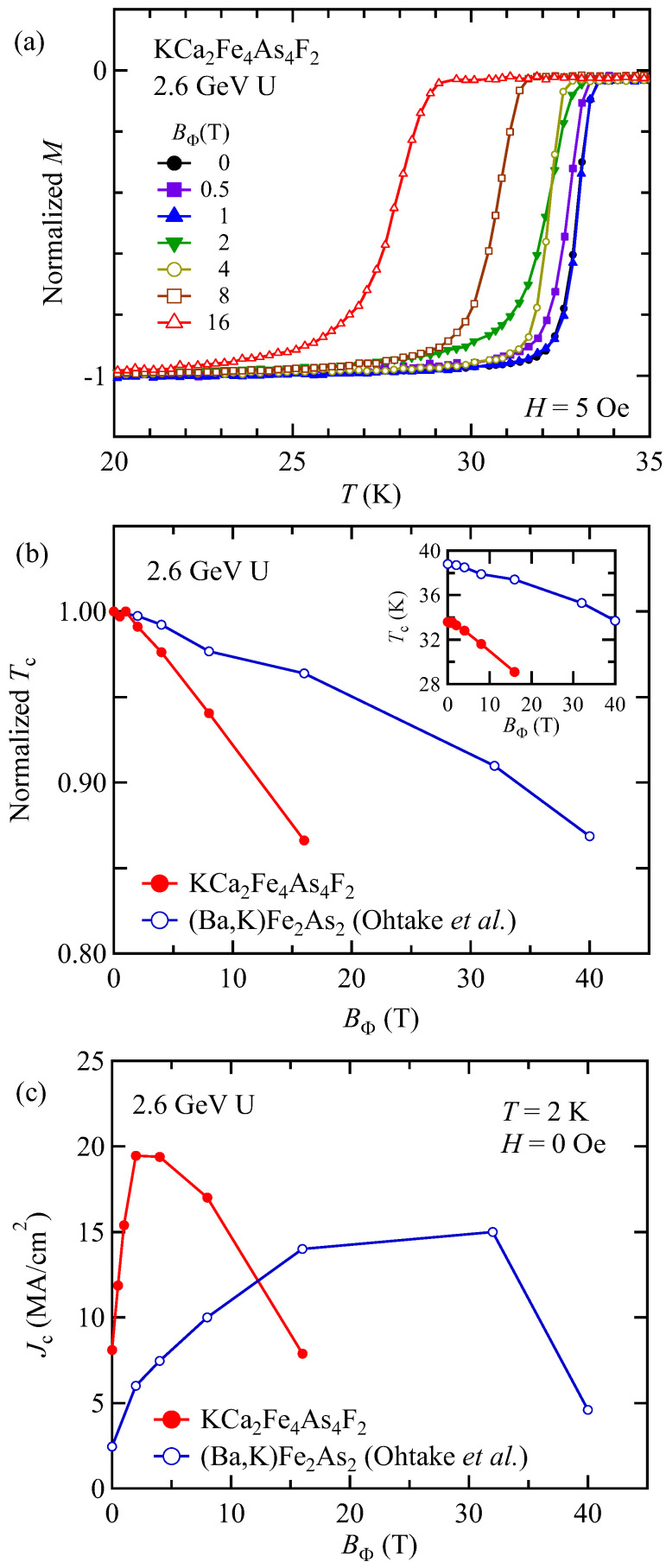
(**a**) Temperature dependence of normalized magnetization of 2.6 GeV U-irradiated KCa_2_Fe_4_As_4_F_2_ with various *B*_Φ_ at 5 Oe. The irradiation dose is evaluated by *B*_Φ_. The *B*_Φ_ dependence of (**b**) normalized *T*_c_ and (**c**) *J*_c_ at 2 K under self-field in 2.6 GeV U-irradiated KCa_2_Fe_4_As_4_F_2_ and (Ba,K)Fe_2_As_2_ [26]. The *B*_Φ_ dependence of *T*_c_ is also shown in the inset of (**b**).

Next, the effects of columnar defects on *T*_c_ and *J*_c_ in KCa_2_Fe_4_As_4_F_2_ were investigated. Figure 2a shows zero-field-cooled magnetization measurements with a *H*//*c*-axis for 2.6-GeV U-irradiated KCa_2_Fe_4_As_4_F_2_. It is clear that the onset of superconductivity is systematically suppressed by increasing *B*_Φ_. Normalized *T*_c_ estimated from the linear extrapolation from the low temperature is summarized in Figure 2b. The *B*_Φ_ dependence of *T*_c_ is also shown in the inset of Figure 2b. For comparison, the *B*_Φ_ dependence of *T*_c_ for (Ba,K)Fe_2_As_2_ are also shown [26]. *T*_c_ of both KCa_2_Fe_4_As_4_F_2_ and (Ba,K)Fe_2_As_2_ are monotonically decreased with increasing *B*_Φ_, although *B*_Φ_ dependence of *T*_c_ in KCa_2_Fe_4_As_4_F_2_ is stronger than that of (Ba,K)Fe_2_As_2_. The rate of the reduction of the normalized *T*_c_ estimated from Figure 2b is 0.3%/T and 0.9%/T for (Ba,K)Fe_2_As_2_ and KCa_2_Fe_4_As_4_F_2_, respectively. If columnar defects only destroy local superconductivity, *T*_c_ should not be affected by introducing such defects. The suppression of *T*_c_ can be explained by considering the effect of point defects created by secondary electrons, which are generated via interaction with highly energetic U ions and the lattice [26]. It is known that magnetic impurities lead to suppression of *T*_c_ in *s*-wave superconductors, as discussed by Abrikosov and Gor’kov [40], and point defects may work similar to magnetic impurities in anisotropic KCa_2_Fe_4_As_4_F_2_. Unfortunately, however, it is not easy to estimate the dose and energy of such secondary electrons in a given material. 

Next, the in-plane *J*_c_ for *H*//*c*-axis in KCa_2_Fe_4_As_4_F_2_ is evaluated by measuring the irreversible magnetization using the extended Bean model [20,41,42],

*J*_c_ [A/cm^2^] = 20Δ*M*/*a*(1 − *a*/3*b*) (1)

where Δ*M* [emu/cm^3^] is *M*_down_ − *M*_up_. *M*_up_ and *M*_down_ are the magnetization when sweeping the field up and down, respectively, and *a* [cm] and *b* [cm] are the sample width and length (*a* < *b*). Figure 2c shows the *B*_Φ_ dependence of *J*_c_ in KCa_2_Fe_4_As_4_F_2_ irradiated with 2.6 GeV U ions at *T* = 2 K under the self-field. For comparison, similar data for a (Ba,K)Fe_2_As_2_ single crystal are also shown [26]. The *B*_Φ_ dependence of *J*_c_ in KCa_2_Fe_4_As_4_F_2_ shows similar trends to that of (Ba,K)Fe_2_As_2_, although the *B*_Φ_ for the largest *J*_c_ are quite different. In the low *B*_Φ_ region, *J*_c_ is enhanced in proportion to *B*_Φ_^−1/2^. This can be understood by a simple estimation based on half-loop excitations of vortices in the matrix of discontinuous columnar defects [26]. By increasing *B*_Φ_ further, *J*_c_ takes a maximum at a certain *B*_Φ_, and it starts to decline above this value. The maximum *J*_c_ of 19.4 MA/cm^2^ is realized in KCa_2_Fe_4_As_4_F_2_ with *B*_Φ_ = 2 T, and that of 15 MA/cm^2^ is realized in (Ba,K)Fe_2_As_2_ with *B*_Φ_ = 32 T. This value is more than twice as large as that of *J*_c_ of the pristine sample, and larger than that of irradiated (Ba,K)Fe_2_As_2_~15 MA/cm^2^, as shown in Figure 2c [26]. The *J*_c_ in KCa_2_Fe_4_As_4_F_2_ starts to decline above *B*_Φ_ = 4 T, which is much earlier than that in (Ba,K)Fe_2_As_2_, as shown in Figure 2c. This early *J*_c_ suppression at a smaller *B*_Φ_ is consistent with stronger suppression of *T*_c_, which could be due to stronger generation of secondary electrons, as discussed above. 

**Figure 3:**
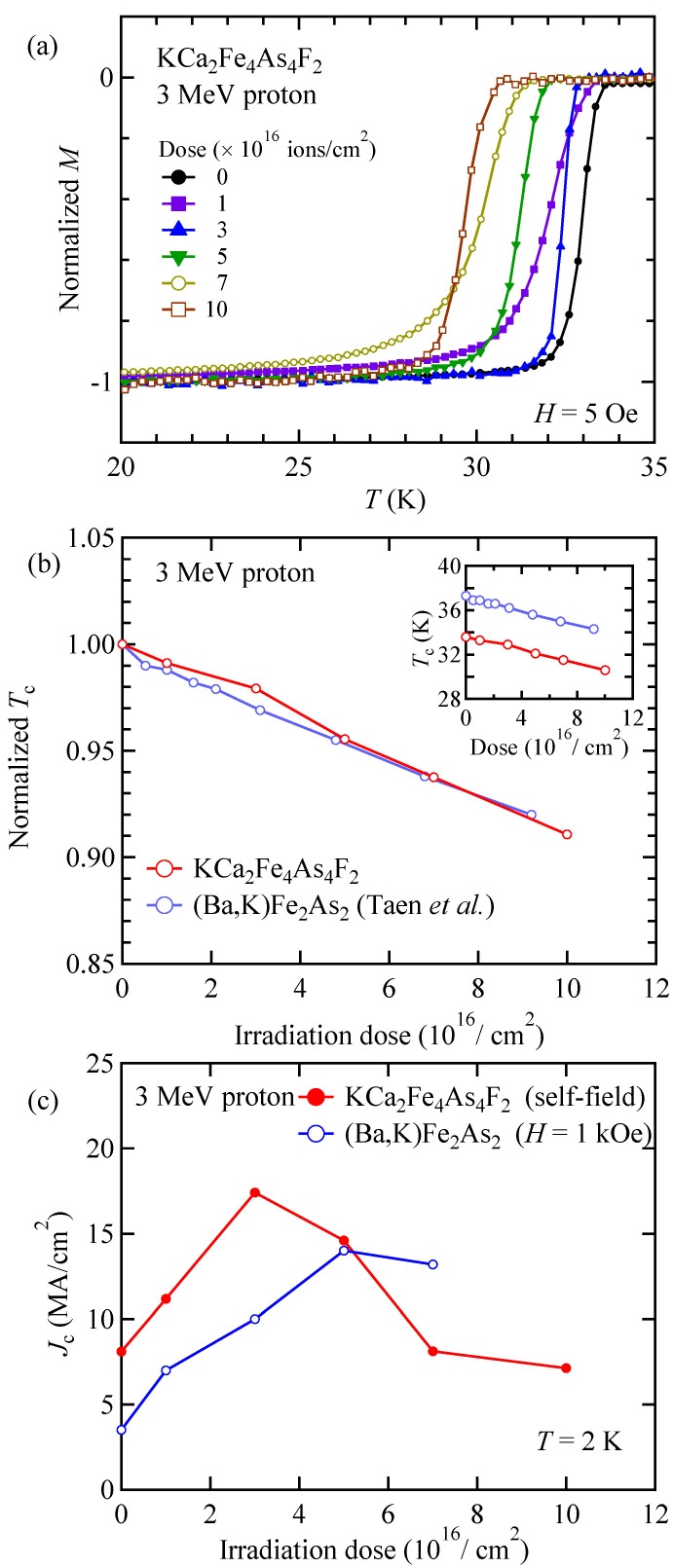
(**a**) Temperature dependence of normalized magnetization of 3 MeV proton irradiated KCa_2_Fe_4_As_4_F_2_ with various dose at 5 Oe. (**b**) Dose dependence of normalized *T*_c_ in 3 MeV proton irradiated KCa_2_Fe_4_As_4_F_2_ and (Ba,K)Fe_2_As_2_ [27]. Dose dependence of *T*_c_ is also shown in the inset of (**b**). (**c**) Dose dependence of *J*_c_ at 2 K in 3 MeV proton irradiated KCa_2_Fe_4_As_4_F_2_ at self-field and (Ba,K)Fe_2_As_2_ at *H* = 1 kOe.

Following the effect of columnar defects, the effects of point defects on *T*_c_ and *J*_c_ in KCa_2_Fe_4_As_4_F_2_ were investigated. Similar to the case of columnar defects, the onset of superconductivity was systematically suppressed by increasing dose, as shown in Figure 3a. Figure 3b shows the dose dependence of normalized *T*_c_ in 3 MeV proton irradiated KCa_2_Fe_4_As_4_F_2_. The dose dependence of *T*_c_ is also shown in the inset of Figure 3b. For comparison, the dose dependence of *T*_c_ for (Ba,K)Fe_2_As_2_ is also shown in Figure 3b and in its inset [43]. The *T*_c_ of both KCa_2_Fe_4_As_4_F_2_ and (Ba,K)Fe_2_As_2_ monotonically decrease with increasing proton dose. It should be noted that the rate of *T*_c_ reduction in both KCa_2_Fe_4_As_4_F_2_ and (Ba,K)Fe_2_As_2_ estimated from Figure 3b are 0.9 and 1.0% per 1 × 10^16^ ions/cm^2^, respectively. These results suggest that the density of created point defects by proton irradiation and their effects on *T*_c_ is similar in KCa_2_Fe_4_As_4_F_2_ and (Ba,K)Fe_2_As_2_. This is in contrast to the case of 2.6-GeV U-irradiated KCa_2_Fe_4_As_4_F_2_ and (Ba,K)Fe_2_As_2_, where the amount of point defects generated by secondary electrons may depend on the material. Figure 3c show the dose dependence of *J*_c_ in KCa_2_Fe_4_As_4_F_2_ irradiated with 3 MeV proton at *T* = 2 K under a self-field. For comparison, similar data for (Ba,K)Fe_2_As_2_ measured at *H* = 1 kOe are also shown. *J*_c_ in KCa_2_Fe_4_As_4_F_2_ reaches its maximum of 17.5 MA/cm^2^ at a dose of 3 × 10^16^ ions/cm^2^. On the other hand, *J*_c_ of (Ba,K)Fe_2_As_2_ reaches its maximum of 14 MA/cm^2^ at a dose of 5 × 10^16^ ions/cm^2^. The doses for maximum *J*_c_ are similar, although that of KCa_2_Fe_4_As_4_F_2_ is a little smaller. Such a difference can be explained by the fact that the pristine KCa_2_Fe_4_As_4_F_2_ contains embedded point defects, which are atomic-scale defects due to strains or chemical inhomogeneities, and are responsible for high *J*_c_ by working as pinning centers [16]. As discussed above, suppression of *T*_c_ and the dose dependence of *J*_c_ in 2.6-GeV U-ion or 3 MeV proton-irradiated KCa_2_Fe_4_As_4_F_2_ are different from those in typical IBS of (Ba,K)Fe_2_As_2_, which can be explained by considering the embedded defects in pristine KCa_2_Fe_4_As_4_F_2_. It should be emphasized that maximum *J*_c_ in KCa_2_Fe_4_As_4_F_2_ is larger than that in 122 compounds, which have been extensively studied as raw materials for wires and tapes for future high-field applications [44,45,46]. Fabrications of wires and tapes of KCa_2_Fe_4_As_4_F_2_ are demanded.

### 3.2. Vortex Dynamics in Pristine and Proton Irradiated KCa_2_Fe_4_As_4_F_2_

**Figure 4:**
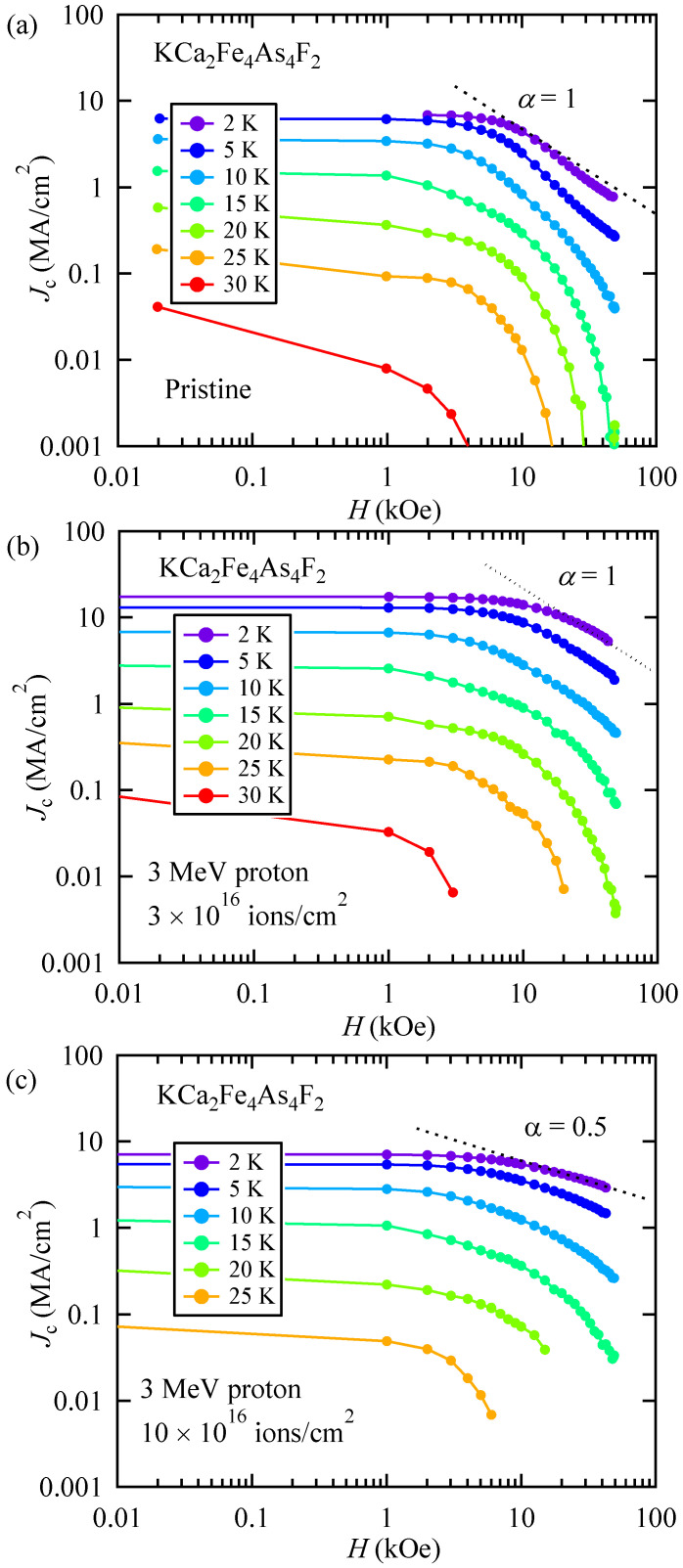
Magnetic field dependence of critical current densities for *H*//*c* at various temperatures in (**a**) the pristine and 3 MeV proton irradiated KCa_2_Fe_4_As_4_F_2_ with dose of (**b**) 3 × 10^16^ or (**c**) 10 × 10^16^ ions/cm^2^.

Figure 4a shows magnetic field dependence of *J*_c_ for *H*//*c* at various temperatures in the pristine KCa_2_Fe_4_As_4_F_2_. At all temperatures, *J*_c_ monotonically decreases with magnetic field. These features are similar to those in optimally-doped (Ba,K)Fe_2_As_2_ [27] and Li_0.8_Fe_0.2_OHFeSe [37]. By contrast, a broad peaks in *J*_c_-*H* curves, sometimes referred to as fish-tail effects, are observed in other IBSs, such as Ba(Fe,Co)_2_As_2_ [24] and Fe(Te,Se) [35]. One of the possible mechanisms of the fish-tail effect is weak collective pinning, which is attributed to the presence of dense atomic-scale defects [47,48]. The absence of a fish-tail effect in KCa_2_Fe_4_As_4_F_2_ is possibly explained by the dominance of strong pinning due to the presence of embedded defects in pristine crystal. Furthermore, at low temperatures below 15 K and above ~10 kOe, *J*_c_ shows a power-law decay with the field, *J*_c_ ∝ *H*^−α^, with *α*~1. The decay of *J*_c_ proportional to *H*^−1^ is observed in YBa_2_Cu_3_O_7_ with columnar defects, which is explained as follows [19]; for example, at *H* = 10 kOe, the distance between vortices is (Φ_0_/*H*)^1/2^~50 nm. If the pinning centers are sparse and their average separation is larger than 50 nm, all these pinning centers are occupied by vortices above 10 kOe. Hence above this field, the pinning force *F*_p_ will stay constant, in spite of the increase in *H*. So, the value of *J*_c_ will decrease in proportional to *H*^−1^, since *F*_p_ = 1/*c*·*J*_c_*H* [49,50]. In other words, observation of *α*~1 suggests that there are sparse strong pinning centers in the pristine KCa_2_Fe_4_As_4_F_2_, which may also explain the exceptionally large *J*_c_ at low temperatures in this system. It may also explain the slightly lower *T*_c_ in KCa_2_Fe_4_As_4_F_2_ compared with (Ba,K)Fe_2_As_2_ with a similar hole number per Fe. On the other hand, at higher temperatures above 15 K, *J*_c_ decreases faster with magnetic field, and no power-law field dependence is observed. Similar behavior is also observed in 3 MeV proton irradiated KCa_2_Fe_4_As_4_F_2_, where *J*_c_ takes its maximum at a dose of 3 × 10^16^ ions/cm^2^. As shown in Figure 4b, at low temperatures below 15 K and above ~20 kOe, *J*_c_ again shows the power-law decay, *H*^−α^ with *α*~1. Above 15 K, *J*_c_ decreases strongly with the magnetic field. This trend is very similar to that in the pristine sample, although the field dependence of *J*_c_ is a little weaker. As shown in Figure 4c, in 3 MeV proton irradiated KCa_2_Fe_4_As_4_F_2_ with a dose of 10 × 10^16^ ions/cm^2^, the power-law field dependence of *J*_c_ with smaller *α* (~1/2) is observed at low temperatures, although such behavior is absent at temperatures above 10 K. With increasing the proton dose, *α* at low temperatures changes from ~1 to ~1/2. In the case of sparse strong pinning, *α* = 1/2 or 5/9 is predicted [48,51]. So, the change of *α* from ~1 to ~1/2 may indicate the change of pinning, from very sparse strong pinning to sparse strong pinning. Vortices in the pristine KCa_2_Fe_4_As_4_F_2_ are pinned by embedded defects. After the proton irradiation, the strong pinning of vortices by point defects introduced by proton irradiation becomes dominant. We have reported changes of *α* from ~1/2 to ~1/3 by creating point defects via proton irradiation in various IBSs [24,27,52,53]. The difference in the field dependence of *J*_c_ between KCa_2_Fe_4_As_4_F_2_ and other IBSs may be caused by the existence of embedded defects in the pristine KCa_2_Fe_4_As_4_F_2_. Another remarkable feature of *M*(*H*) is fast decay of *J*_c_ at higher temperatures and higher magnetic fields. Such behavior of *J*_c_ may be related to highly anisotropic properties in KCa_2_Fe_4_As_4_F_2_, which will be discussed below. 

**Figure 5:**
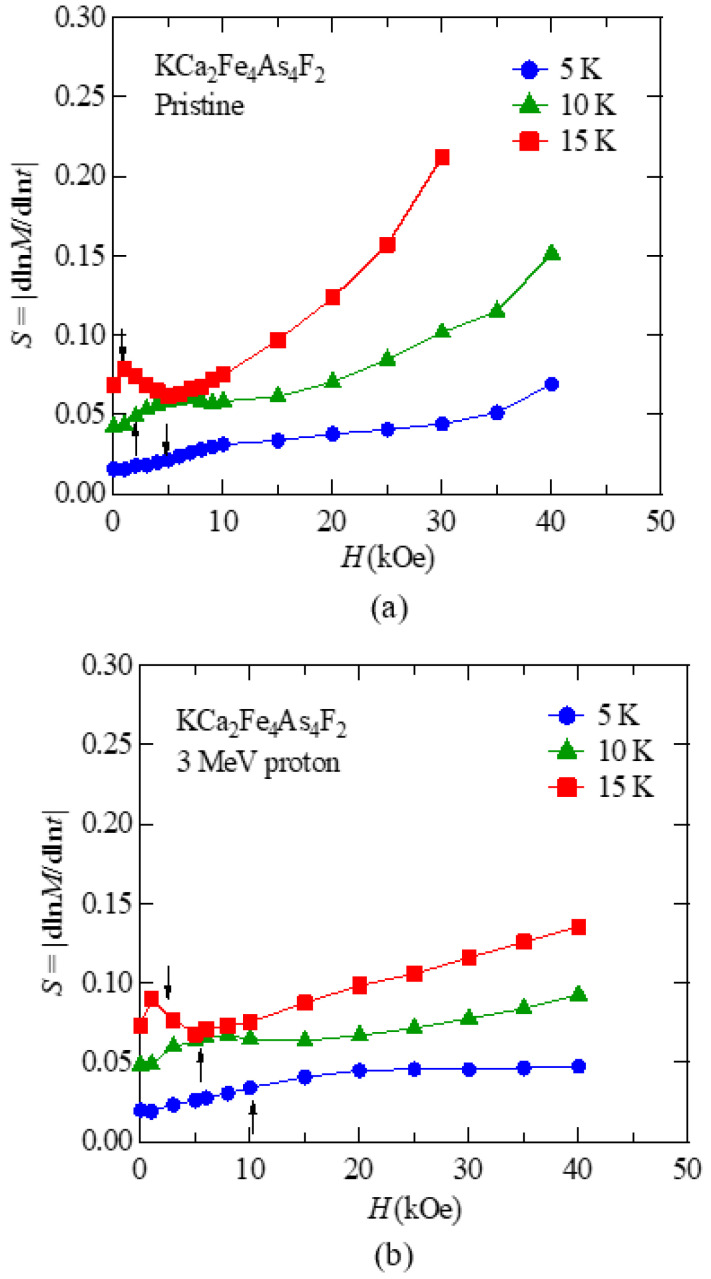
Magnetic field dependence of the normalized magnetic relaxation rate *S* = |dln*M*(*t*)/dln*t*| at *T* = 5, 10, and 15 K under *H*//*c* in (**a**) the pristine and (**b**) 3 MeV proton irradiated (3 × 10^16^ ions/cm^2^) KCa_2_Fe_4_As_4_F_2_. The self-fields at 10 and 15 K of both the pristine and proton irradiated samples are indicated by arrows.

Vortex dynamics in the pristine and proton irradiated KCa_2_Fe_4_As_4_F_2_ crystal were investigated by measuring the normalized magnetic relaxation rate, *S* = |dln*M*(*t*)/dln*t*|. The decay of magnetization with time, *M*(*t*), due to creep motion of vortices was traced for an hour, after the critical state was prepared. Figure 5a,b show the magnetic field dependence of *S* at *T* = 5, 10, and 15 K for *H*//*c* in the pristine and 3 MeV proton irradiated KCa_2_Fe_4_As_4_F_2_ at a dose of 3 × 10^16^ ions/cm^2^, respectively. The dimensions of the pristine and proton irradiated samples were 0.0178 × 0.0212 × 0.0008 and 0.0235 × 0.049 × 0.0013 cm^3^, respectively. There were three notable features in the behavior of *S*. First, characteristic dip features at low magnetic fields below 10 kOe could be identified in both the pristine and 3 MeV proton irradiated KCa_2_Fe_4_As_4_F_2_. The characteristic field for the suppression of *S* is roughly equal to the self-field *H*_sf_ = *J*_c_·*t*, as we discussed in Ref. [54], where *t* is the thickness of the sample, as indicated by arrows. *H*_sf_ values for the pristine sample were evaluated as 4.7 kOe, 2.0 kOe, and 0.7 kOe at 5 K, 10 K, and 15 K, respectively, while those for the proton-irradiated sample were evaluated as 10.5 kOe, 5.3 kOe, and 2.2 kOe at 5 K, 10 K, and 15 K, respectively. Similar suppressions of *S* at low magnetic fields below the self-field have been observed in pristine Ba(Fe,Co)_2_As_2_ and (Ba,K)Fe_2_As_2_ [24,27]. Second, at high magnetic fields, *S* in the pristine KCa_2_Fe_4_As_4_F_2_ increases rapidly above a temperature dependent characteristic field, although *S* in the proton-irradiated KCa_2_Fe_4_As_4_F_2_ increases monotonically up to 40 kOe. Third, the values of *S* in KCa_2_Fe_4_As_4_F_2_ are larger than those in (Ba,K)Fe_2_As_2_ and Ba(Fe,Co)_2_As_2_ with values of 0.02–0.03 [24,27]. 

**Figure 6:**
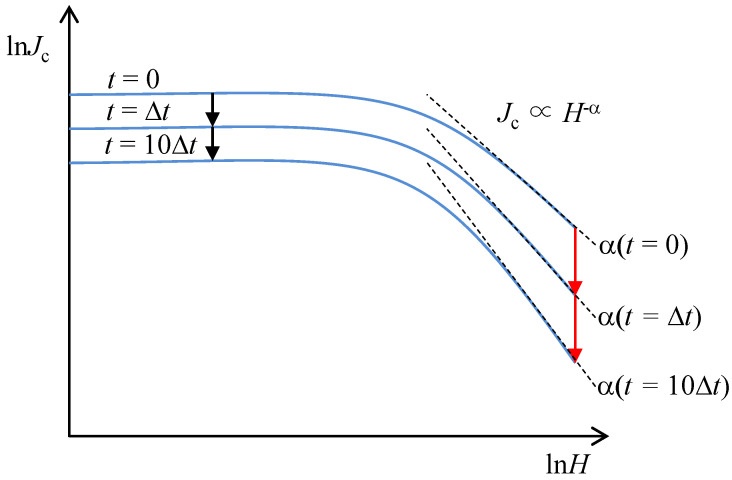
Schematic magnetic field dependence of *J*_c_ in a double logarithmic plot at different times after preparing the critical state at *t* = 0. When the normalized magnetic relaxation rate increases appreciably with the field, measured *J*_c_ values at high fields after some time delay (*t*, 10*t*, …) become smaller, resulting in stronger apparent magnetic field dependence of *J*_c_, namely a larger *α*.

Now, we show that *α*, which determines the field dependence of *J*_c_ (∝*H*^−α^), can be overestimated in KCa_2_Fe_4_As_4_F_2_ due to a large and field-dependent *S*(*H*). This is explained conceptually in Figure 6. When the magnetic field is applied and critical state is formed at *t* = 0, magnetic relaxation starts right after that. On the other hand, magnetization measurements for the evaluation of *J*_c_ are done after some time delay *t*. If *S*(*H*) does not depend on a magnetic field, reduction of *J*_c_, expressed by black arrows, is constant at all fields. In the case of KCa_2_Fe_4_As_4_F_2_ with a field-dependent *S*(*H*) (d*S*(*H*)/d*H* > 0), however, reduction of *J*_c_ with time becomes larger at higher fields, as expressed by red arrows. Such an increased reduction of *J*_c_ at high fields makes apparent *α* larger, which may explain the relatively large *α* in KCa_2_Fe_4_As_4_F_2_. 

Next, we discuss the behavior of *S*(*H*) in low and high magnetic fields. According to the collective creep theory, the *H* dependence of *S* is determined by the variation of the glassy exponent *μ*, as observed in YBa_2_Cu_3_O_7_ [55]. In this theory, the glassy exponent (*μ*) is related to the vortex-bundle size [33]. In a three-dimensional system, it is predicted as *μ* = 1/7, 5/2, 7/9 for single-vortex, small-bundle, and large-bundle regimes, respectively [33,56]. The collective creep theory is characterized by the *J* dependence of *U*. Collective creep theory, which considers vortex elasticity, predicts an inverse power law form for the energy barrier:

(2) $$U\left(J\right)=U_{0}{\left(J_{c0}/J\right)}^{\mu },$$

where *J*_*c*0_ is the temperature-dependent critical current density in the absence of flux creep, *U*_0_ is the flux activation energy in the absence of flux creep, and *U*(*J*) is flux activation energy [57]. This formula can be applied when relaxation process goes on and *J* is reduced (*J* ≪ *J*_*c*0_). On the other hand, at low temperatures and fields, the simple linear relationship proposed in the Anderson–Kim model [33], as shown below, is often accurate:

(3) $$U\left(J\right)=U_{0}\left(1-J_{c0}/J\right).$$

This model neglects vortex elasticity and vortex–vortex interactions. This relation is often limited to the early stages of the relaxation process (*J*~*J*_*c*0_). To express behavior of *U*(*J*) for a wide range of *J*, described by Equations (2) and (3), the interpolation formula is commonly used, as shown below [57]:

(4) $$U\left(J\right)=\frac{U_{0}}{\mu }\left[{\left(J_{c0}/J\right)}^{\mu }-1\right].$$

On the other hand, from the Arrhenius relation, *U* can be also described as:

(5) $$U=k_{B}T\ln\left(t/t_{\mathrm{eff}}\right),$$

where *k*_B_ is the Boltzmann constant and *t*_eff_ is the effective hopping attempt time [34]. By combining Equations (4) and (5), time and temperature dependent *J*(*T*,*t*) and *S* are given as follows [57]:

(6) $$J\left(T,t\right)=\frac{J_{c0}}{{\left[1+\left(\mu k_{B}T/U_{0}\right)\ln\left(t/t_{\mathrm{eff}}\right)\right]}^{1/\mu }}$$

(7) $$S=\frac{k_{B}T}{U_{0}+\mu k_{B}T\ln\left(t/t_{\mathrm{eff}}\right)}.$$

From this formula, we can observe that *S* is enhanced in the single vortex regime, where *μ* takes a relatively small value of 1/7. On the other hand, the density of vortices is relatively sparse at fields below *H*_sf_. In such a low-field regime, vortices may behave independently. So, one of the possible origins of the peak in *S*(*H*) at low magnetic fields is the enhanced *S*(*H*) with a smaller *μ* in the single vortex regime. It should be noted that, in the case of proton irradiated (Ba,K)Fe_2_As_2_, peaks in *S*(*H*) disappear and only dip structures are observed, and *α* changes from 1/2 to 1/3 [27]. From these facts, enhancement of contribution of strong pinning by introducing point defects in (Ba,K)Fe_2_As_2_ is suggested [27]. On the other hand, the degree of suppression of *S* in KCa_2_Fe_4_As_4_F_2_ after proton irradiation is small and *α* is still larger than 1/2. Comparing the effect of proton irradiation on *S*(*H*) at low magnetic fields in these two materials, it is suggested that strong pinning nature of vortices in KCa_2_Fe_4_As_4_F_2_ is even more dominant after proton irradiation compared with that in (Ba,K)Fe_2_As_2_. This is consistent with the change of *α* from 1 to 1/2, as discussed above. At high magnetic fields, *S*(*H*) gradually increases with increasing *H*. As discussed above, one of possible explanations for the increase in *S*(*H*) is gradual reduction of *μ* caused by the change in the vortex bundle size, from small bundles (*μ* = 5/2) to large bundles (*μ* = 7/9). Another possible origin of gradual increase in *S*(*H*) with increase *H* is the significantly low *H*_irr_ in KCa_2_Fe_4_As_4_F_2_ originated from highly anisotropic crystal structure and resulting large anisotropy [12,16]. *S*(*H*) is expected to diverge as *H* approaches *H*_irr_, and *H*_irr_ is reduced with increasing temperature. So, the strong anisotropy in KCa_2_Fe_4_As_4_F_2_ significantly affects the behaviors of *S* at high temperatures and high magnetic fields. It should be pointed out that this *S*(*H*) behavior affects the magnetic field dependence of *J*_c_. At high magnetic fields and high temperatures, a simple power-law dependence of *J*_c_ on *H* is not observed and *J*_c_ decrease rapidly with increasing *H* as shown in Figure 4a–c. This is related to the rapid increase of *S*(*H*) with increasing *H*, as discussed in Figure 6. After the proton irradiation, field dependences of both *J*_c_ and *S* become moderate, as shown in Figure 4b,c and Figure 5b. Although the true *H*_irr_ defined by the onset of nonlinearity in KCa_2_Fe_4_As_4_F_2_ may not be affected by the introduction of point defects, as reported in YBa_2_Cu_3_O_7_ [31], the enhancement of pinning force by proton irradiation explains weaker field dependence of *J*_c_ and *S*.

**Figure 7:**
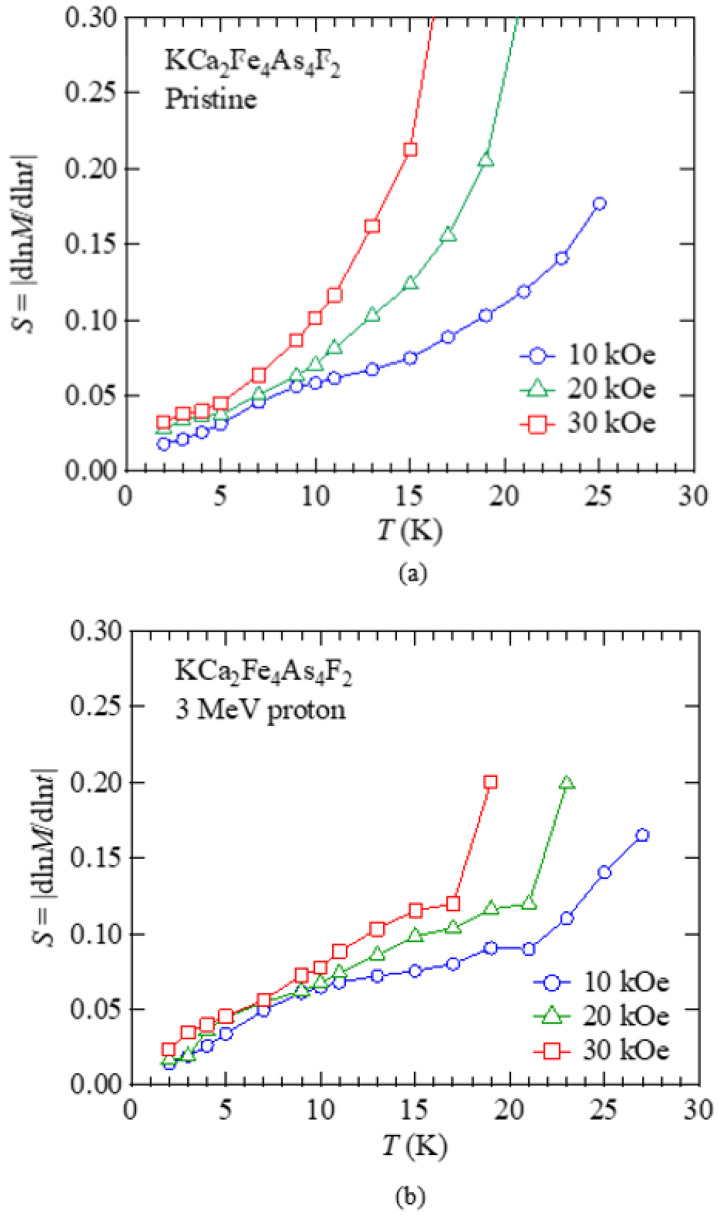
Temperature dependence of the normalized magnetic relaxation rate *S* at *H* = 10, 20, and 30 kOe under *H*//*c* in (**a**) the pristine and (**b**) 3 MeV proton irradiated (3 × 10^16^ ions/cm^2^) KCa_2_Fe_4_As_4_F_2_.

Figure 7a,b show the temperature dependence of *S* at *H* = 10, 20, and 30 kOe for *H*//*c* in the pristine and 3 MeV proton irradiated KCa_2_Fe_4_As_4_F_2_ at a dose of 3 × 10^16^ ions/cm^2^, respectively. In both the pristine and proton irradiated samples, *S*(*T*) shows a monotonic increase with increasing temperature without a plateau-like behavior, and the slope of *S*(*T*) increases with increasing magnetic field. The temperature dependence of *S*(*T*) becomes weaker only at 10 kOe between 10 K and 15 K. According to the collective creep theory, *S*(*T*) is proportional to temperature at low temperatures, while it shows a plateau at intermediate *T* (≫*U*_0_/(*k*_B_ln(*t*/*t*_eff_)) with a value of *S* = 0.02–0.04 [58]. So, one may say that the behavior of *S*(*T*) at 10 kOe in the above-*T* range can be explained in the framework of collective creep theory. However, the value of *S*(*T*) between 10 K and 15 K is larger than the expected value. Plateau-like behaviors of *S*(*T*) have been observed in, not only YBa_2_Cu_3_O_7_ [58], but also in 122-type compounds with reasonable values of *S* of 0.01–0.04 [24,27]. However, with an increasing magnetic field, the weak temperature dependence of *S*(*T*) between 10 K and 15 K disappears, and *S*(*T*) strongly increases at higher temperatures. Low *H*_irr_ in anisotropic KCa_2_Fe_4_As_4_F_2_ can explain the stronger temperature dependence of *S*(*T*) at high fields. On the other hand, such a divergent behavior of *S*(*T*) at high temperatures is suppressed by proton irradiation, as shown in Figure 7b. 

It is important to determine the value of *μ* in discussing vortex dynamics since *μ* includes information on the size of the vortex bundle in the collective creep theory. To extract this value, it is convenient to evaluate inverse current density dependence of effective pinning energy, U^*^, which is defined as follows:

U^*^ = *k*_B_*T*/*S*. (8)

From this equation and “interpolation formula”, U^*^ can be calculated as:

(9) $$U^{*}=U_{0}+\mu k_{B}T\ln\left(t/t_{\mathrm{eff}}\right)=U_{0}{\left(J_{c0}/J\right)}^{\mu }.$$

**Figure 8:**
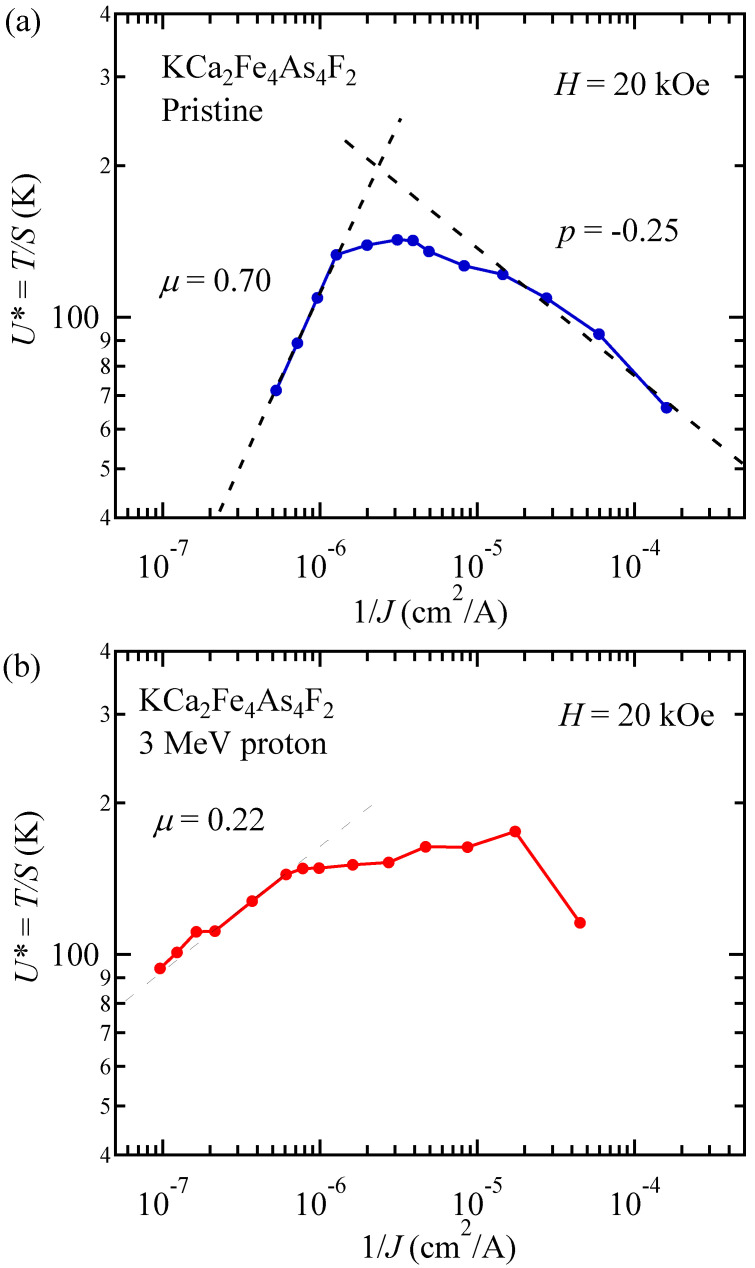
Inverse current density dependence of effective pinning energy U^*^ at *H* = 20 kOe in (**a**) the pristine and (**b**) 3 MeV proton irradiated (3 × 10^16^ ions/cm^2^) KCa_2_Fe_4_As_4_F_2_.

**Figure 9:**
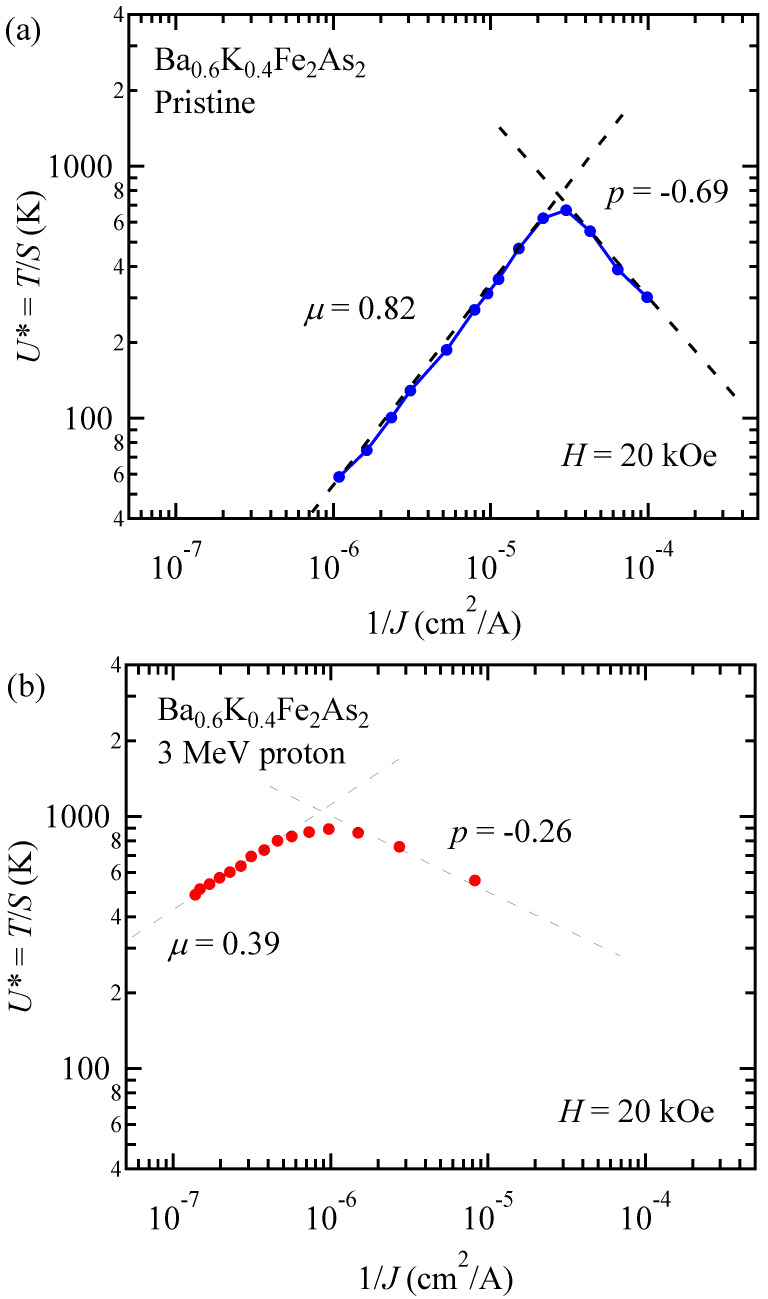
Inverse current density dependence of effective pinning energy U^*^ at 20 kOe in (**a**) the pristine and (**b**) 3 MeV proton irradiated (5.6 × 10^16^ ions/cm^2^) (Ba,K)Fe_2_As_2_.

Thus, the slope in the double logarithmic plot of U^*^ vs. 1/*J* gives the value of *μ*, as shown in Figure 8a,b. For this analysis, a proper choice of *H* is important to determine a region with a unique *μ* [27]. However, since power-law field dependence of *J*_c_ is broken down in KCa_2_Fe_4_As_4_F_2_, we chose a magnetic field of *H* = 20 kOe, where *α* is nearly constant below 15 K to avoid the effect of fast relaxation at high temperatures and at high magnetic fields. In this way, we evaluated *μ* = 0.70 and 0.22 for the pristine and proton irradiated KCa_2_Fe_4_As_4_F_2_, respectively. For comparison, we also plotted U^*^ vs. 1/*J* curves for the pristine and proton irradiated (Ba,K)Fe_2_As_2_ in Figure 9a,b, respectively, using the data of relaxation analyses described in Ref. [27]. We evaluated *μ* = 0.82 and 0.39 for the pristine and 3 MeV proton irradiated (5.6 × 10^16^ ions/cm^2^) (Ba,K)Fe_2_As_2_, respectively. Note that *μ*~1 in pristine crystal is often reported in YBa_2_Cu_3_O_7_ [32] and IBSs [24,59]. After proton irradiation, however, *μ* becomes significantly smaller in both KCa_2_Fe_4_As_4_F_2_ and (Ba,K)Fe_2_As_2_. This trend has also been reported in Ba(Fe,Co)_2_As_2_ [24]. Values of *μ* in various IBSs are summarized in Table 1. The values of *μ* in most of pristine IBSs are ~1, except for Li_0.8_Fe_0.2_OHFeSe. In Li_0.8_Fe_0.2_OHFeSe, a very large value of *μ*~4.1 was reported, although the anisotropy parameter is comparable to that in KCa_2_Fe_4_As_4_F_2_ [37]. It is discussed that the very large *μ* in Li_0.8_Fe_0.2_OHFeSe may indicate that vortices in this material are in the crossover regime between elastic Abrikosov vortices to stacks of pancake vortices. The fact that the *μ* value in KCa_2_Fe_4_As_4_F_2_ was ~1 may suggest that vortices in this material were more like Abrikosov vortices, similar to the case of (Ba,K)Fe_2_As_2_. On the other hand, negative slopes of U^*^ versus 1/*J* are also observed at small *J*. This negative slope is often denoted as *p* in the plastic creep scenario with *p*~−0.5 and is confirmed experimentally [60]. Evaluated *p* = −0.25 and −0.69 for pristine KCa_2_Fe_4_As_4_F_2_ and (Ba,K)Fe_2_As_2_ are roughly consistent with this scenario. In the case of proton-irradiated KCa_2_Fe_4_As_4_F_2_, however, we were unable to access the negative slope region at a small *J*, while *p* = −0.26 was obtained in the proton-irradiated (Ba,K)Fe_2_As_2_. Reduction of |*p*| after introduction of point defects was also observed in Ba(Fe,Co)_2_As_2_ [24]. Dose dependent measurements of vortex dynamics in the plastic region may shed light on the evolution of the plastic behavior.

From the above experimental results and discussion, we can form a conjecture that predominant pinning in the pristine KCa_2_Fe_4_As_4_F_2_ is as follows. The large *J*_c_~8 MA/cm^2^ in the pristine sample is attributable to the embedded strong pinning centers, while artificial defects introduced by the irradiation enhance *J*_c_ to more than double. Vortex dynamics in KCa_2_Fe_4_As_4_F_2_ is similar to those in weakly anisotropic (Ba,K)Fe_2_As_2_ rather than Li_0.8_Fe_0.2_OHFeSe with similar anisotropy parameters. However, the effects of proton irradiation on magnetization, magnetic relaxation, and their behaviors at high temperatures and high magnetic fields are different from those in (Ba,K)Fe_2_As_2_. Low *H*_irr_ in KCa_2_Fe_4_As_4_F_2_ due to large anisotropy causes a breakdown of clear power-law dependence of *J*_c_ on the magnetic field. Since the slope of U^*^ − 1/*J* curve shows negative slope in a wide range of 1/*J* in the pristine sample, the effect of the plastic creep should be considered for the comprehensive understanding of the vortex dynamics in this system. The small value of *H*_irr_ and the large value of *S*, as well as the rapid decay of *J*_c_-*H* at high temperatures and high magnetic fields, support this idea. We leave it as an open problem since the study on such fast dynamics is beyond the scope of this paper.

## 4. Summary

*J*_c_ and vortex pinning properties in KCa_2_Fe_4_As_4_F_2_ single crystals before and after introduction of artificial defects by ion irradiations are systematically studied. Columnar and point defects are introduced by 2.6-GeV U-ions and 3 MeV protons irradiations, respectively. The in-plane *J*_c_ evaluated from the measurement of irreversible magnetization at 2 K under the self-field is 8 MA/cm^2^, which is the largest among all IBSs. *J*_c_ under self-field at 2 K is strongly enhanced up to 19.4 or 17.5 MA/cm^2^ by irradiation of 2.6 GeV U-ions or 3 MeV protons, respectively. In both cases, suppression of *T*_c_ is observed. Quantitative differences of irradiation dose dependence of *T*_c_ compared with those of (Ba,K)Fe_2_As_2_ can be explained by considering the presence of embedded defects in the pristine KCa_2_Fe_4_As_4_F_2_. Vortex dynamics in the pristine and proton irradiated KCa_2_Fe_4_As_4_F_2_ single crystals are also investigated from the analyses of field dependence of *J*_c_ and the normalized magnetic relaxation rate. The values of *α* from ~1 to ~1/2 before and after proton irradiation, and the behavior of *S* suggest that the vortex system in the pristine and proton irradiated KCa_2_Fe_4_As_4_F_2_ can be described by strong pinning. Vortex dynamics in KCa_2_Fe_4_As_4_F_2_ is similar to that in weakly anisotropic (Ba,K)Fe_2_As_2_ rather than Li_0.8_Fe_0.2_OHFeSe with similar anisotropy parameter. Low *H*_irr_ in KCa_2_Fe_4_As_4_F_2_ due to large anisotropy can explain some of unique behaviors of *J*_c_ such as break down of clear power-law dependence on the magnetic field.

## Figures and Tables

**Table 1:** Glassy exponents for elastic creep *μ* in various IBS compounds, estimated from U* vs. 1/*J* plots.

	KCa_2_Fe_4_As_4_F_2_	(Ba,K)Fe_2_As_2_	Ba(Fe,Co)_2_As_2_	Li_0.8_Fe_0.2_OHFeSe	Fe(Te,Se)	FeSe
Pristine	0.70	0.82	1.09 [24]	4.1 [37]	1.34 [35]	0.71 [36]
Proton liubinirradiated	0.22	0.39	0.82 [24]	-	-	-

## Data Availability

The data presented in this study are available on request from the corresponding author.
